# In-service teachers’ perceptions of project-based learning

**DOI:** 10.1186/s40064-016-1725-4

**Published:** 2016-01-26

**Authors:** Anita Habók, Judit Nagy

**Affiliations:** Institute of Education, University of Szeged, Petőfi S. sgt. 30-34, Szeged, 6722 Hungary; Department of English Language Teacher Education and Applied Linguistics, University of Szeged, Szeged, Hungary

**Keywords:** In-service teachers’ perceptions, Project-based learning, Traditional classroom instruction

## Abstract

The study analyses teachers’ perceptions of methods, teacher roles, success and evaluation in PBL and traditional classroom instruction. The analysis is based on empirical data collected in primary schools and vocational secondary schools. An analysis of 109 questionnaires revealed numerous differences based on degree of experience and type of school. In general, project-based methods were preferred among teachers, who mostly perceived themselves as facilitators and considered motivation and transmission of values central to their work. Teachers appeared not to capitalize on the use of ICT tools or emotions. Students actively participated in the evaluation process via oral evaluation.

## Background

Project-based learning (PBL) is a learning method based on constructivism (Hmelo-Silver 2004), which was first proposed by John Dewey at the end of the 1890s (Douglas and Stack 2010). Dewey’s philosophy was child-centred and introduced real-life situations and contexts into the school environment. His ideas were further developed by Kilpatrick in the early 1900s in his book The Project Method (1918). Since then, PBL has been elaborated in detail and applied to various school subjects and learning situations. As a result of such practical applications, our understanding of PBL has been greatly enriched. In more recent research, Hovey and Ferguson (2014) have pointed out that there are different interpretations of PBL with various overlapping terms, for example, problem-based learning, inquiry-based learning, problem learning and the project method. In addition, activity-based learning and discovery learning encompass similar features. Previously, Holm (2011) defined PBL in a practice-based manner as “student-centred instruction that occurs over an extended time period, during which students select, plan, investigate and produce a product, presentation or performance that answers a real-world question or responds to an authentic challenge” (Holm 2011, p. 1). According to a previous, but more comprehensive definition, PBL is “a systematic teaching method that engages students in learning knowledge and skills through an extended inquiry process structured around complex, authentic questions and carefully designed products and tasks” (Markham et al. 2003, p. 4). While the former definition relies on student-centred learning processes, the latter also places emphasis on the development of skills in addition to knowledge acquisition and the crucial task of planning, including task design and the complexity and authenticity of questions. A comparison of these two definitions sheds light on some of the issues underlying ongoing discussions on the definition of PBL.

After a review of the variety of available definitions and approaches, a much needed comprehensive summary of the characteristics of PBL has been provided by Markham et al. (2003). In their approach, the fundamental criteria for PBL include: student’s drive to learn; a focus on student-centred processes; familiarizing students with the core concepts in disciplines and topics; focus questions to enable in-depth exploration; students’ management of their own work and projects; outcomes related to students’ problem-solving and investigations; provision of feedback; emphasis on student cooperation in small groups through student presentations and class evaluation; application of modern ICT tools; performance-based assessment; and the incorporation of PBL into the curriculum. These criteria establish the foundation for a standards-based project framework which increases performance and accountability (Markham et al. 2003, pp. 4–5).

However, significantly fewer studies have addressed teachers’ views on the efficacy of this method, the differences compared to traditional classroom activities and the ways in which teachers can capitalize on opportunities provided by 21st-century innovations. In this novel context, competitiveness can only be ensured by enhancing our knowledge and acquiring learning skills which enable us to adapt to societal changes and requirements. Value appears in the form of knowledge generated via intellectual means, not only in the form of material products. Knowledge that is acquired and applied mechanically is ephemeral; thus, new approaches are necessary and creativity assumes a greater role. Moreover, a distinction has to be made between two types of knowledge, which often do not overlap, namely, classroom knowledge and practical knowledge, which is an everyday necessity and a regular expectation on the labour market. Similarly, good communication, excellent problem-solving skills and the ability to work individually and in a team are also among the most frequent requirements in job advertisements. Consequently, the development of such skills should be included in the curriculum. Bell claims that PBL is not merely a supplementary activity to boost learning, but a fundamental part of the curriculum and that PBL involves a science-based approach and the development of skills, which would also be necessary for learning situations in general (Bell 2010). In order to accomplish this goal, applied methods, such as PBL, should develop these skills in addition to sharing information.

## Theoretical framing of PBL

### PBL as an educational approach for learning and teaching

Traditional education and standardized testing generally fail to comply with 21st-century requirements, which consist of the external requirements of the workplace and internal requirements, such as individual learner needs. In addition, there is a wide array of learners, including low-achieving students and students with special educational needs, whose instruction requires innovative methods. This is emphasized by Thomas (2000), who reports on the effectiveness of PBL in diverse contexts, including racially diverse groups and low-achieving students. Oakley et al. (2004) have proposed preventing at-risk minority students from becoming isolated by forming groups of three or four students representing diverse ability levels. Moreover, they suggest using the Team Policies Statement and the Team Expectations Agreement, which helps groups establish rules that all members can adhere to. In addition, it provides a framework for dealing with problems that might arise during the project (Oakley et al. 2004).

Careful planning is necessary to implement a successful project. Indeed, in contrast with traditional methods, both teachers and learners engage in the planning process in PBL. The increased burden of planning is likely to result in increased responsibility and independence on the part of learners, as they have to plan their own activity and set the goals they will need to accomplish either individually or in groups. Setting goals strengthens accountability for individuals and groups as well. Peer pressure works as a regulatory force that is often more powerful than the teacher’s requests, as social ties with peers are likely to be more close-knit and thus instil a greater degree of motivation. This is an aspect which deserves more attention in testing PBL project outcomes (Bell 2010). Oakley et al. (2004) also stress the importance of peer evaluation. They distinguish two types of peer evaluation. The first covers evaluation of the individual work of participants in relation to the outcome of the product, and the second entails social aspects, such as cooperation, management, supportiveness and tolerance, which they refer to as “team citizenship”. The first approach fosters academic achievement and competitiveness, and consequently disfavours low-achieving students, who might lack the necessary skills and perceive the obstacles as insurmountable and as a result are likely to lose motivation. The second approach is based on teamwork and social skills rather than academic performance, and, as such, provides an incentive to low-achieving students to actively participate because their efforts will be reflected in their grades even if they are not able to perform on a par with high-achieving students (Oakley et al. 2004).

At present, there is a compelling need for the measurement of skills such as communication, self-evaluation, negotiation, cooperation, collaboration and tolerance in PBL. Since self-evaluation has become an essential skill in the 21st century, PBL projects need to address the self-evaluation of learning and social skills, which are indispensable for lifelong and lifewide learning in a knowledge-based society. Present and future members of the workforce are evaluated not only on their professional accomplishments, but also on the processes of organization, in-group communication and negotiation. Thus, social skills are becoming increasingly important (Bell 2010).

### Key components of PBL

As stated previously, Dewey’s work on student engagement and practice-based learning are essential components of PBL. Since students’ voice is required during the phases of the project, the significance of student voice is addressed in curriculum theory (Cook-Sather 2010). Student voice is generally considered to be a significant element of the learning process. However, in Hungarian education the traditional approach prevails and teachers take centre stage, controlling and directing the learning process (Doró and Balla 2014). Learners are mostly motivated externally and are not required to carry out individual or collaborative work which fosters learner motivation and autonomy. In contrast, PBL is a student-driven process, which is only facilitated but not controlled by teachers. By focusing on the solving of real-life problems, PBL helps learners become autonomous learners (Bell 2010).

Besides cognitive constructivism, the role of social constructivism is emphasized, since learning occurs in a social environment. Vygotsky (1978, cited in Jarvis 2005) stated that students’ social skills have to be fostered by group work and cooperation. Bandura (1977) stressed the defining role of peers in the development of social skills, for example, the beneficial effect of observing a good example, which can serve as a basis for imitation of learning behaviour. Metacognitive and cooperative skills, cooperation and creativity are essential for problem-solving and learning in the 21st century. Moreover, metacognition, higher-order thinking and reflective practice play a significant role in the evaluation and success of the learning process (Hovey and Ferguson 2014; Holm 2011).

Hovey and Ferguson (2014) summarized the key components of PBL based on Thomas (2000) and the Buck Institute of Education (BIE 2013). Firstly, the curriculum is centred on a complex project, which is constructed around a focus question. Secondly, learner initiative is essential on both the individual and cooperative levels of the project. Their definition suggests a pragmatic approach, as learner motivation is enhanced by real-world application in everyday contexts. Finally, reflective evaluation and revision are continuous with a final evaluation based on the final outcome of the project (BIE 2013; Thomas 2000; Trilling and Fadel 2009). Reflectivity and critical thinking play a central role during the research stage of PBL and the real-world application of knowledge. Learners need to be able to judge the adequacy and reliability of the information they encounter, for example, while they use ICT tools to research their project topics. Teachers need to provide guidance on the safest and most efficient methods and techniques for internet research (Bell 2010).

A further key component of PBL is interdisciplinarity, which includes the in-depth study of specific topics and establishes relationships between various subjects. As a result, students are not restricted by the boundaries of traditional subject areas and are enabled to establish relationships between kinds of information pertaining to different subjects. For example, they are able to learn about the history, art, music and literature of the Renaissance period in a comprehensive manner, resulting in a complex set of knowledge (Kalyoncu and Tepecik 2010).

### The project as a process

The planning and implementation of a project is a highly time-consuming activity and requires great attention to detail. There are numerous aspects which call for careful consideration (Habók 2015). Firstly, choosing a topic and a title that is to the point is very important. Involving students in the decision-making process is beneficial because they will feel more involved in the project on the whole. In addition, student involvement in selecting the topic, which may cover one subject or can be interdisciplinary, is also key. Increased engagement results in a greater number of shared experiences and thus facilitates motivation. Secondly, planning involves assigning roles and activities, organizing groups, and establishing venues and financial and time requirements. During the planning stage, teachers should consider the features of the venue and ensure that groups have sufficient workspace without distracting each other. Moreover, all participants should be able to accomplish the task, and the necessary tools should be available to everyone. At this stage, the teacher controls the process, but students may also be involved. Klug et al. (2014 full abstract) highlighted the importance of teacher behaviour and its long-term effect on lifelong learning. Their research revealed a greater amount of teacher success in sparking interest in a new topic than supporting students during the planning process.

In addition, the project should ensure that students carry out research and work cooperatively in order to enhance their problem-solving skills, motivation and creativity. Data collection may take place within or outside the classroom. Students can carry out research within the classroom using available literature and online resources, or they can expand their learning environment and gather information in a wider context, for example, by organizing trips. The topic can be discussed during regular lessons, or separate days can be allocated exclusively to the project. In Hungary, it is common practice to organize projects weeks at the end of the term.

Finally, evaluation focuses on the presentation of the final product, which can take various forms, such as a school presentation, a short film, a diary entry or any other form which helps students summarize the work process. The final presentation also necessitates planning, as students need to agree on the roles and tasks of each participant prior to the presentation. Evaluation may take various forms; besides teacher evaluation, peer and self-evaluation are also available. Since PBL departs from the traditional classroom approach, evaluation should be devised accordingly. Traditional evaluation methods most probably are not suitable for the measurement and assessment of the knowledge and skills acquired during PBL. More fitting evaluation methods include peer evaluation, self-evaluation, oral presentation and a practical exam (Habók 2015). Although PBL projects are generally short-term projects ranging from a few days to few months in duration, Thomas reports the beneficial effect of the use of PBL over a span of 3 years, with a significant increase in performance (Thomas 2000).

### Previous research projects

Campbell (2012) observed the use of PBL in ESL classrooms with 15–16-year-old students. The study used mixed methods including observations and a collection of artifacts, direct instruction times and attendance. During the analysis of over 60 h of observation, various themes were identified, including direct instruction, missing directions, wasted time, computer distractions, attendance, follow-through, vocabulary instruction, grouping, class size, percentage of ELL students, student motivation, use of resources, differentiated instruction, and student confidence and ability. It was concluded that the development of communicative competences enhanced collaboration (Campbell 2012).

Language teaching and intercultural education offers yet another context in which the notion of authenticity is foregrounded. Real-life applications and authentic materials are especially relevant for language learners, who otherwise would not encounter such contexts in their everyday lives. Presenting cultural and social aspects of language learning was a central goal in the PBL project implemented by Wu and Meng (2010). They emphasized that PBL facilitated the acquisition of such knowledge even for learners with low language proficiency. The development of communicative competence and the accomplishment of communicative goals were fostered by cooperation and ‘learning by doing’. This development was also clearly visible in the posttest scores of the experimental group, who were seen to be more motivated than the control group. The benefits of PBL were observed in the development of cognitive and metacognitive strategies and the increase of motivation, which resulted in an increase in English proficiency. During the evaluation of the programme, learners reported increased intercultural knowledge, highly positive attitudes towards PBL and increased cultural sensitivity, motivation and language proficiency. Moreover, development of metacognitive skills was also reported. Furthermore, enhancing English proficiency and communicative competence also involves pronunciation teaching. Metacognitive skills and metalinguistic awareness can be developed by using visualization tools in pronunciation teaching, especially with the use of ICT tools in Computer Assisted Pronunciation Teaching (Nagy 2014).

Hallerman et al. (2011) defined the essential elements of PBL categorized into two main groups: significant content and 21st-century skills. Significant content consists of three elements, driving question, in-depth inquiry and public audience, and focuses on teaching subject-based knowledge and skills. 21st-century skills include the need to know, student voice and choice, revision and reflection, skills which facilitate critical thinking, problem solving, collaboration and cooperation, and communication. According to Hallerman et al. (2011), a successful project is based on meaningful learning and authentic tasks and products, student discovery and real-world application.

The involvement of real-life application and real-world objects in interdisciplinary science projects produces considerably improved learner experience and frequently results in increased motivation even in heterogeneous groups involving low-achieving students. In a qualitative study, Baumgartner and Zabin (2008) analysed the effect of students’ scientific knowledge and attitudes. The results demonstrated the positive effect of PBL on students’ understanding of scientific processes and attitudes. Similarly, Beneke and Ostrosky (2008) examined teacher perceptions and revealed a positive view on the part of teachers and increased motivation among learners, including differently-abled learners, who also benefitted from PBL. They also reported the positive effect of involving real-world objects in the preschool world. These results also show that PBL can cater to a variety of learner types. These results were supported by Cheng et al. (2008), who also demonstrated that PBL is effective in heterogeneous groups as well, since it was group processes, and not the structure of the group, that were identified as predictors of self-efficacy, irrespective of the performance level of learners. Chu et al. (2011) used mixed methods and combined inquiry PBL and the collaborative teaching method with Year 4 students and found that information literacy and ICT skills developed. Similarly, Grant and Branch (2005) carried out a PBL-based project to map individual differences and abilities and found evidence for the flexibility of this method, which was used in various contexts. Doppelt (2003) maintains that the use of PBL reinforced motivation and positive self-concept among low-achieving students in 3 years of training, thus improving their performance during that time. Throughout the programme, students developed their metacognitive skills by solving interdisciplinary problems and managed their own work, while documenting the steps of the process. Real-life applications were intertwined with the original goal of the electricity track. In addition to professional skills, students developed their ICT skills while researching the topic and documenting implementation. Furthermore, Duncan and Tseng (2010) applied PBL in biology and concluded that concept learning was successful, however not to the level that had been hypothesized.

In general, teachers have the role of facilitator and guide, and they can also provide scaffolding in PBL. Several studies have focused on effectiveness in PBL (Holm 2011; Bell 2010).

Teacher guidance is commonly claimed to be important in the implementation of PBL. However, this is difficult to accomplish unless the teachers receive adequate training. Besides theoretical training, teachers also need practical training to be able to fully exploit the potential of this method (Wu and Meng 2010). Tal et al. (2006) recorded examples of good practice and identified teacher skill as a predictor of the success of PBL. In addition, other key contributing factors were suitable curriculum materials and teacher content knowledge. Hertzog (2007) has mentioned difficulties in the implementation of PBL with Year 1 students, stemming from the beliefs of teachers concerning teaching methods and children’s needs. At the outset, other types of instruction were preferred due to time requirements. School policies and curriculum requirements also hindered the implementation of projects. However, the final results pointed to a more student-centred approach and increased engagement. Balasubramanian et al. (2014) measured the perceptions of 249 students on the use of a learner-centred ICT learning environment and found that students preferred this platform for the management of learning via forums and development of social skills. However, in other contexts, it was noted that the responsible and critical use of ICT tools posed extra tasks for the teacher in terms of planning and management of class activities (Campbell 2012).

## Methods

### Research design

In the present study, we analysed teachers’ voice with regard to PBL. The survey was designed for elementary and secondary school teachers, who were requested to complete the online questionnaire containing 15 questions, including five questions about their background. Participation was optional, and completion of questionnaires took about 20 min and was carried out at the participant’s convenience. Schools were contacted via telephone to ensure that all respondents actually used PBL in their teaching practice. Direct confirmation from each educational institution was necessary, as not all schools use PBL and it is occasionally confused with cooperative learning or problem-based learning. As the first step of the recruitment procedure, school principals were contacted and were entrusted with the decision whether to forward the questionnaire to teachers in their institution. The email invitation included detailed information regarding the aim of the study and confidentiality issues.

### Participants

In total, teachers from eight schools completed the questionnaire. By ensuring anonymity, we aimed to encourage teachers to complete the questionnaire in a forthcoming and honest way without fear of inquiries or repercussions concerning their work. A total of 109 questionnaires were completed. The effect of school type is also addressed, as the sample includes 32 teachers in Years 1–4 and 42 in Years 5–8 in primary schools, and 35 teachers in vocational school and/or vocational secondary school. Both vocational schools and vocational secondary schools offer learners various forms of training, but only learners in vocational secondary schools enjoy the option of taking Matura examinations, which grant them access to tertiary education. In terms of teaching experience, the sample included a wide array of teachers. During the analysis, we included 34 teachers in the group of *beginner teachers*, who have 10 or fewer years of practice, 30 teachers in the group of *experienced teachers* having 11–20 years of practice, and 44 teachers in the group of *expert teachers*, who have more than 21 years of practice.

### Measures

The questionnaire was self-developed and preceded by a small-scale pilot study (n = 33). The topics addressed in the present study had been established based on teacher interviews. The first part of the questionnaire includes five background questions (gender, age, teaching experience, and type and location of school). In the subsequent analysis, we address teaching experience and type of school as central factors. In the following section of the questionnaire, we set out to examine teachers’ perceptions and beliefs regarding teacher and student participation in PBL and traditional classroom instruction. Firstly, effectiveness of teaching methods and classroom management are addressed. Teachers were requested to select their preferred alternatives from a list of 17 items. Next, they were asked to choose the most important teacher roles in project work and traditional classroom teaching. Afterwards, the three most important characteristics of a successful project and a successful traditional classroom lesson had to be identified by teachers based on a list. Teachers were also expected to provide information on the subjects involved in the project. Teachers were likewise asked about student participation in the evaluation process within traditional classroom activities and project work.

### Aims and research questions

The main objectives of the present research was to analyse teachers’ teaching methods and to discover teachers’ opinions about PBL. Our research questions are the following:

1. Which teaching methods are used frequently?
2. What is the teacher’s role in PBL? What is the teacher’s role in traditional classroom teaching?
3. What are the characteristics of a successful project? What are the characteristics of a successful classroom lesson?
4. Do students take part in evaluation? If yes, which method(s) can/do they use? What evaluation methods are applied during traditional classroom teaching and learning? Do students participate in the evaluation during or at the end of the project?

## Results

### Frequently used methods

The first question concerns the most frequently used teaching methods. Our results show that teachers generally use demonstration (52 %) and work-based learning (46 %), but group-based methods (39 %), the cooperative method (37 %), PBL (36 %) and games (33 %) are also used. On the other hand, student presentations (11 %), discussions (9 %) and lectures (5 %) are rarely employed.

**Fig. 1:**
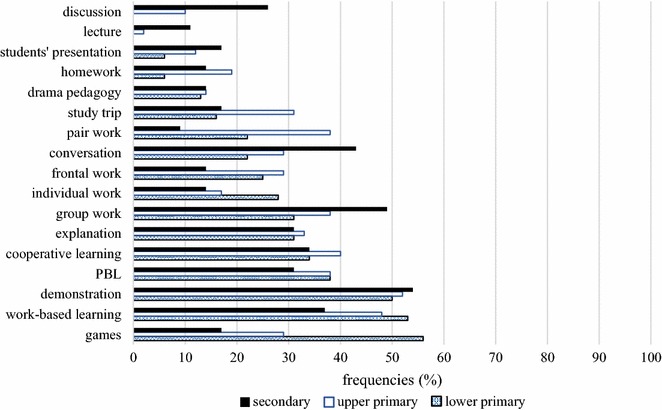
Teachers’ responses about frequently used teaching methods

Next, we examined the relationship between the level of the school where teachers work and their preferred methods. We analysed teachers’ answers according to three groups: (1) lower primary school teachers, (2) upper primary school teachers and (3) secondary school teachers (Fig. 1).

In general, PBL is among the favoured methods in all three groups, yet it is not the most frequent at any level. Lower primary school teachers prefer games (56 %), work-based learning (53 %) and demonstration (50 %), followed by PBL (38 %). Upper primary school teachers use demonstration (52 %) most commonly and then work-based learning (48 %) and cooperative learning (40 %). These methods are followed by PBL, which, similarly to lower primary school, is used by 38 % of teachers. The distribution is different in secondary school, where 14 % of teachers use frontal work, which is the dominant method. Other methods include demonstration (54 %) and conversation (43 %). In addition, lecture and discussion are not used in lower primary school and are rare in upper primary school (lecture 2 % and discussion 10 %). Similarly, in secondary school, lectures (11 %) and pair work (9 %) are rarely used. In line with our results, Le Fevre (2014) maintains that teachers tend to avoid risks associated with changing their teaching practices. They prefer teacher-centred education because they strive to maintain control. Taking on the role of facilitator requires great effort because they often assume that this role entails losing control over classroom activity. Adherence to the traditional textbooks is one example of risk avoidance, and the associated fear of losing control is especially dominant concerning the use of ICT tools, where students might possess a wider array of skills and knowledge and even outperform teachers.

Subsequently, we analysed data according to teaching experience to map potential differences between beginner, experienced and expert teachers with regard to their use of methods. Teachers were grouped into three categories: (1) *beginner teachers* with less than 10 years of experience; (2) *experienced teachers* with 11–20 years of experience; and (3) *expert teachers* with over 20 years of experience. Results show that on the whole, the most frequently used methods are demonstration (Freq._1–10 years_ = 56 %, Freq._11–20 years_ = 53 %, Freq._>20 years_ = 50 %) and work-based learning (Freq._1–10 years_ = 44 %, Freq._11–20 years_ = 40 %, Freq._>20 years_ = 50 %). Our teachers emphasized the importance of work-based learning. Similarly, Alake-Tuenter et al. (2013) have confirmed that inquiry-based teaching and work-based teaching represent significant teaching competences. The third most frequently used method among beginner teachers and expert teachers is group work (Freq._1–10 years_ = 35 %, Freq._>20 years_ = 45 %); it is conversation (50 %) for experienced teachers. It is not surprising that the least experienced teachers report using drama pedagogy (6 %) the least frequently, since this method requires substantial experience and most probably can only be implemented during a separate course. On the other hand, the expert teachers used this method more extensively (Freq._>20 years_ = 25 %). A further distinction is that expert teachers to not use lecture at all, whereas 12 % of beginners do. It is likely that they do not have an extensive methodological repertoire and prefer relying on this teacher-centred method, since it equips them with more control over the class. However, lecture as a frontal method often only provides an illusion of joint progress as individual thoughts and opinions are not revealed.

Despite the fact that a number of researchers have confirmed the effectiveness of the use of ICT tools in PBL (Doppelt 2003; Chu et al. 2011; Balasubramanian et al. 2014), our results show that secondary school teachers mostly use frontal instruction and do not make use of the availability of ICT tools. We believe that this is problematic because students in this age group are highly involved in the uses of ICT tools in everyday contexts and thus are likely to be susceptible to such media in educational settings as well. As a result, we believe that teachers should capitalize on ICT tools in the classroom to a greater extent.

#### Teacher’s role in PBL

The second research question focused on the teacher’s role in the project method. Our initial assumption was that teachers were aware of the fact that their role had shifted to that of a facilitator, instead of a more traditional role. In line with these assumptions, results show that most teachers assign importance to motivation (63 %), transmission of values (40 %) and forming personality (34 %). Throughout traditional instruction motivating learners (57 %) is considered most important, along with teaching (51 %) and transmission of values (43 %). It is worth noting that teaching, social and affective education and maintaining discipline are linked more closely to traditional instruction and not to PBL. The acquisition of skills also takes place in PBL, yet teachers might find it more difficult to monitor it as compared to traditional teaching situations.

**Fig. 2:**
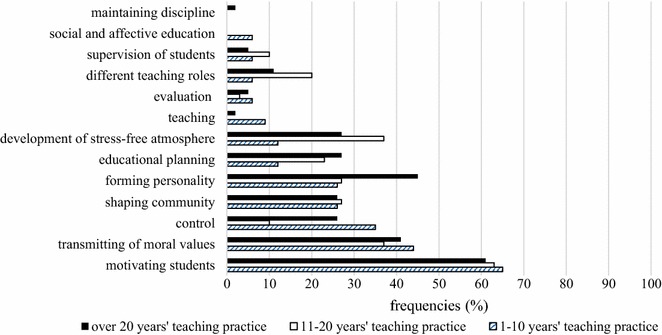
Teachers’ responses about their role in PBL

Teacher and school-related factors also reveal a certain variation in teacher perceptions. The comparison of teachers based on school types reveals that motivation is important in PBL, according to more than half of the teachers. In addition, transmission of values and forming personality are also considered important. As revealed by examining results according to teaching experience, over 60 % of teachers highlight the importance of motivation and transmission of values as teacher roles. Expert teachers deem forming personality (45 %) more important than other teachers. Maintaining discipline is not considered important at all by this group; that is, they do not position themselves in a controlling role. The underlying reason might be that they are well aware of the flexible and collaborative nature of project work, which is incompatible with the requirements of frontal work, such as sitting quietly and listening to the teacher (Fig. 2).

Surprisingly, teachers do not necessarily see themselves in the role of the instructor, educator or assessor in PBL. Although only a low number of teachers see themselves as evaluators, it must be noted that this role is very important throughout teaching, as teachers need to be able to assess the effectiveness of their own methods besides the evaluation of students. Moreover, according to teacher perceptions, the teacher’s role in maintaining discipline is less significant. In contrast, motivating students is considered an essential teacher role. The effect of PBL on motivation has been demonstrated by previous research (Doppelt 2003; Filippatou and Kaldi 2010; Hung et al. 2012).

The use of PBL met with a favourable response, and learners were able to engage in academic communicative activities in the collaborative processes of project work, which increased their motivation from the beginning of the project (Campbell 2012). Similarly, Wu and Meng (2010) witnessed the advantage of PBL in the fostering of cognitive and metacognitive strategies and the enhancement of motivation. These factors promoted the development of English language proficiency and cultural sensitivity. Cognitive skills were also developed with young learners with PBL. Habók (2015) reports the successful implementation of a concept map-based PBL developmental programme among pre-school children. The outcome of the programme was increased experimental reasoning and comprehension as compared to the control group. The positive effect of PBL on learning was also measured in later ages. For example, Bagnasco et al. (2010) analysed the effect of PBL on the academic performance of nursing students and found that problem-based learning embedded in PBL promoted learning.

Furthermore, teachers’ self-perception and conceptualization of teacher roles have a fundamental impact on the teaching process. Beneke and Ostrosky (2008) analysed PBL from the teachers’ perspective and its beneficial effect on learners. They found that different types of learning could be developed. One advantage of PBL is that it can cater to the needs of differently-abled learners and all of them can find achievable tasks which still pose a challenge for them. In addition, several researchers have emphasized the role of teachers as facilitators and guides (Holm 2011; Bell 2010).

#### Teacher roles in traditional instruction

**Fig. 3:**
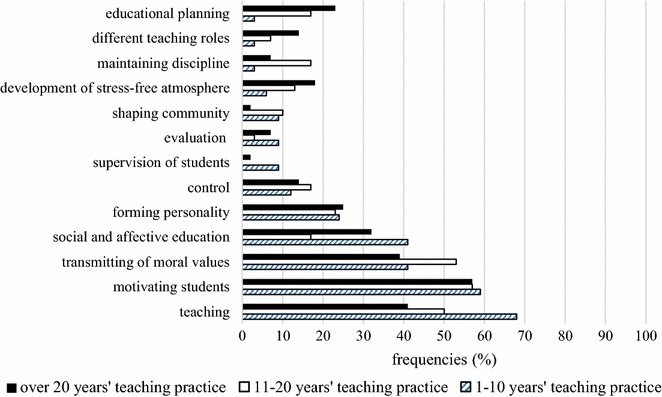
Teachers’ responses about their role in a traditional classroom situation

In the second part of our second research question, we set out to examine teacher perceptions regarding teacher roles during traditional classroom management. Within the bounds of traditional instruction, teaching (Freq._1–10 years_ = 68 %, Freq._11–20 years_ = 50 %, Freq._>20 years_ = 41 %) took centre stage, especially for less experienced teachers. One possible explanation is that they are more focused on curriculum requirements and standardized tests, which do not measure students’ ability to cooperate. Furthermore, motivation was viewed as an important task for teachers (Freq._1–10 years_ = 59 %, Freq._11–20 years_ = 57 %, Freq._>20years_ = 57 %), besides transmission of values (Freq._1–10 years_ = 41 %, Freq._11–20 years_ = 53 %, Freq._>20 years_ = 39 %). According to teacher perceptions, less important teacher roles included control, evaluation and shaping community (Fig. 3), which is a difficult feat to accomplish in a less stable context, where groups frequently change and mostly frontal work is employed. In addition, evaluation is included in the curriculum and as such is carried out by teachers nevertheless.

#### Characteristics of successful projects in PBL

Our third research question dealt with the characteristics of successful projects. Teachers claimed that student activity (62 %) and good atmosphere (59 %) are the key characteristics of a successful project. Similarly, these two features were mentioned for traditional teaching situations, where student activity (51 %), good atmosphere (43 %), enthusiastic learners (43 %) and varied methodology (36 %) were considered essential for success. Although the use of modern ICT tools is inseparable from education, the use of such tools (5 %) was not considered important in PBL, and even in traditional instruction only 17 % of teachers noted it as a key component. Evaluation was also relegated to the background in PBL. In fact, evaluation did not take the form of marks gained for their performance in distinct sections of the project; instead, peer and teacher evaluation is available throughout the implementation of the project.

**Fig. 4:**
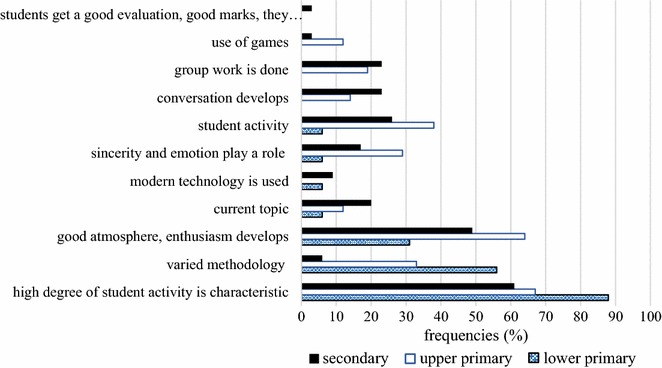
Teachers’ responses about the characteristics of a successful project

In traditional classroom situations, we find a lower degree of consideration of honesty and emotion (6 %) than in PBL, where 18 % of teachers note it. In today’s digital culture, increased attention should be paid to addressing questions related to the interpretation and consideration of other people’s feelings, along with the expression of students’ own feelings. Zembylas et al. (2014) looked into the relationship between emotion and memory in the classroom using the concept of emotional styles. They examined the regulative effect of emotional style on what was considered relevant and what was retained and reiterated in the classroom with regard to a controversial historical event in Cyprus. They emphasize the need for pedagogical strategies to help teachers to manage emotions and memories in the classroom (Zembylas et al. 2014, p. 78). According to our results, upper primary school teachers found it important to address the issue of emotion in the classroom (Fig. 4).

Furthermore, we examine the opinions of teachers operating at different levels of the educational system. At each level, learner activity is considered essential (Freq._lower prim._ = 88 %, Freq._upper prim_. = 67 %, Freq._second_. = 61 %). One surprising result is that varied methodology is deemed important by 56 % of lower primary school teachers, 33 % of upper primary school and merely 6 % of secondary school teachers, even though a differentiated use of methods is strongly justified by diverse learner needs. As far as good atmosphere is concerned, it is viewed as important mostly by upper primary school teachers (64 %), followed by secondary school teachers (49 %), while lower primary school teachers (31 %) consider it less important. On the whole, opinions among upper primary and secondary school teachers were more diverse, while lower primary school teachers mostly agreed on the importance of student activity, varied methodology and good atmosphere.

On the other hand, teaching experience accounts for a wider variation. Beginner and expert teachers believe that good atmosphere (Freq._1–10 years_ = 65 %, Freq._>20 years_ = 61 %), and motivating learner activity (Freq._1–10 years_ = 35, Freq._>20 years_ = 50 %) are most important; however, experienced teachers also point to learner activity as a key factor (Freq._11–20 years_ = 70 %, Freq._>20 years_ = 61 %).

In conclusion, we maintain that projects require a considerable amount of preparation and planning, but the benefits are indisputable. The question of planning and preparation was also examined by Gillies and Boyle (2010), who presented teachers’ experience with cooperative learning. Their analysis showed that teachers gained valuable experience using PBL, but they also encountered a number of difficulties. Both cooperative learning and PBL require a great deal of preparation and planning, with an increased focus on time management. Moreover, assessment posed an additional challenge, as they could not use traditional assessment methods. It was highlighted that cooperative teaching entails thorough planning, planning challenging tasks and laying the groundwork for the monitoring of group activities.

#### Characteristics of success in traditional instruction

Teachers in general agree on the fact that traditional classroom instruction is characterized by a high degree of learner activity; 43 % of expert teachers agreed. Besides learner activity, varied methodology (43 %) is noted by experienced teachers. The importance of games emerged, since students enjoy games, which can spark interest in learners, especially lower primary school students, who frequently engage in autonomous games. Despite this, only 6 % of beginner teachers and 10 % of more experienced teachers state that games are central elements of successful traditional lessons. Likewise, only 11 % of the most experienced teachers consider games important. Moreover, it is even less preferred in PBL.

**Fig. 5:**
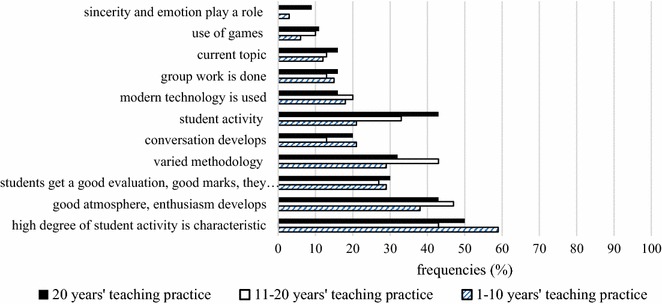
Teachers’ responses about the characteristics of traditional classroom teaching

Moreover, it should also be noted that the role of emotions was neglected by teachers in general. A mere 3 % of beginner teachers and 9 % of expert teachers consider it important, while none of the experienced teachers do. These opinions are particularly perplexing because students study several subjects involving emotional aspects, including literature, art and music. In fact, it is indeed difficult for teachers to be attentive and open to their students’ emotions during frontal work. This problem could be resolved with the use of pair or group work, which makes it possible for learners to communicate and discuss their feelings. However, only 15 % of beginner teachers, 13 % of experienced teachers and 16 % of expert teachers consider pair and group work an effective tool for a successful lesson (Fig. 5). This is in line with research highlighting that group work may often be employed without thorough planning regarding group composition, the quality of the activity, and problem-solving and conflict resolution. Empirical results have demonstrated that teachers may face difficulties during cooperative learning due to its complexity, and, as a result, increased emphasis on professional development is required in this area (Gillies and Boyle 2010, p. 938).

One positive outcome of our research is that it reveals that teachers deem student activity important. This is a favourable attitude, since students will need to be able to engage in active learning and manage their own learning in their future learning processes. Finsterwald et al. (2013) summarized learning-to-learn competences, which include: teacher knowledge regarding the enhancement of students’ LLL competences, such as motivation, self-regulated learning and social and cognitive competences; teacher beliefs in fostering LLL; and teacher motivation, including teachers’ occupational motivation and personal and collective self-efficacy (Finsterwald et al. 2013, p. 148). On the whole, these competences are fundamental, not only in PBL but also in traditional instruction.

### Student evaluation

**Fig. 6:**
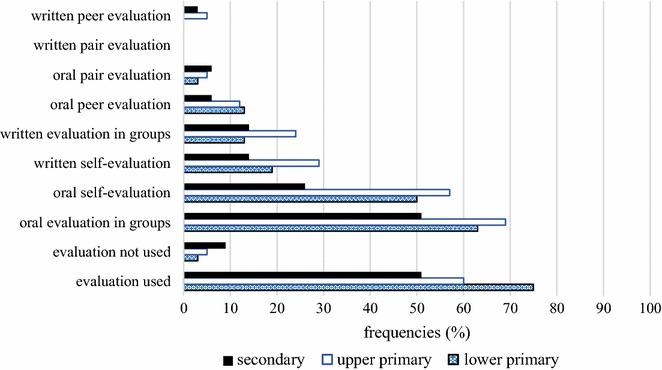
Teachers’ responses about children’s participation in evaluation during the project

Our final research question focused on students’ participation in the evaluation process during or at the end of the project. Surprisingly, some teachers do not involve students in evaluation at all. Unfortunately, this approach is merely favoured by 6 % of teachers in PBL and 18 % in traditional classroom instruction. In PBL, oral evaluation in groups is the most frequent method, as 61 % of teachers use it, followed by oral self-evaluation (45 %). These two types of evaluation are the most popular in the traditional setting as well, where oral self-evaluation (56 %) is the most common, along with group evaluation (32 %). Moreover, these types of evaluation are most frequently used in upper primary school (Fig. 6).

After examining the responses according to the type of institution, we note that oral evaluation in groups is used by the majority of lower primary school teachers (63 %) and it is the most popular approach in upper primary (69 %) and secondary school (51 %) as well. Oral self-evaluation is frequently used in lower (50 %) and upper primary school (57 %), but rarely in secondary school (26 %). A clear distinction can be seen in the case of oral pair evaluation, written pair evaluation and written peer evaluation, which are seldom used on any educational level (Fig. 6).

**Fig. 7:**
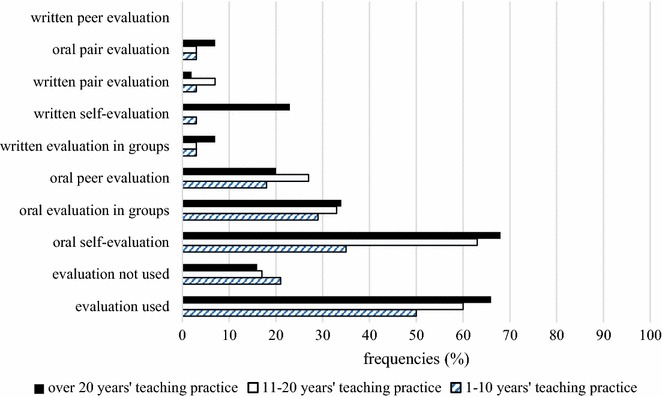
Teachers’ responses about children’s participation in a traditional classroom situation

We find a similar distribution when we examine teacher responses according to teaching experience (Fig. 7). It is the most experienced teachers that involve students in the evaluation process to the greatest degree. However, one possible disadvantage of peer evaluation may be that it is highly time-consuming and teachers are likely to find it impossible to allocate time to lengthy evaluation sessions, especially when they are constrained by a demanding curriculum. Unfortunately, this disadvantage appears to outweigh the interpersonal benefits of enabling learners to form a tighter bond with their classmates. Lee and Lim (2012) list such benefits of peer evaluation, which, in their view, facilitates interaction between learners and generates more heterogeneous student evaluations. However, student feedback mostly addresses social skills, management and coordinating managerial abilities, and not cognitive performance. Nonetheless, involving students in the evaluation process is advantageous because it involves active participation, not merely the passive reception of information.

According to our results, evaluation is frequently used in the traditional setting as well, albeit not at all educational levels. For example, 37 % of secondary school teachers do not involve their students in the evaluation process. In this case as well, oral self-evaluation is predominant. On the other hand, written peer evaluation and pair evaluation are seldom employed or not at all, despite the acclaim with which they have met in previous research. Harris and Brown (2013) have carried out research on peer and self-assessment, focusing on improvement, accountability, social interaction and accuracy. They have concluded that students necessitate guidance during the implementation of peer and self-assessment, for example, in the form of clear instructions. However, they conducted case studies with merely three teachers and thus their results cannot be generalized. Yet they still provide in-depth insights into the application of peer and self-assessment. Nevertheless, it has to be noted that their views are based on the assumption that evaluation is not as central to student progress as it is considered in certain countries, for example, in the UK, the US and China.

Furthermore, Harris and Brown (2013) have identified positive student attitudes towards teacher-controlled assessment. Peer assessment was difficult to implement, since the task of assessment was almost exclusively attributed to teachers. It was suggested that besides training on assessment techniques, supportive psychological concepts such as self-regulation also need to be presented to students to enhance effectiveness. In addition, these efforts need to be backed by policies, which at this point mostly support examinations and teacher-centred evaluation (Harris and Brown 2013, p. 110).

A further advantage of teacher-controlled assessment stems from its greater objectivity as compared to student assessments, which are characterized by increased subjectivity. Van den Bergh et al. (2006) note that students may not like peer assessment due to its subjective nature, which students may exploit to evaluate each other more positively or negatively than their actual achievement based on social rather than academic factors. As a result, students prefer combining such assessment with methods that allow for more objectivity. A further result was that students found self-assessment problematic due to the subjectivity and incomparability of grades. Its benefits mostly reside in mapping group dynamics and self-reflectivity (Van den Bergh et al. 2006, p. 358). Furthermore, students who participated in the research project listed several benefits, including being able to participate in actual fieldwork and apply different types of knowledge in a practical manner over an extensive period of time. Instructors reported increased student motivation, and both mentioned the advantages of undertaking collaborative and individual work as well. Finally, PBL also provides opportunities for student–teacher collaboration (Van den Bergh et al. 2006, pp. 353–354).

## Summary and conclusion

PBL has undergone significant development in the past 30 years as compared to the ideas proposed by Dewey (Douglas and Stack 2010) and Kilpatrick (1918). Learning theories, such as cognitive learning theory and social learning theory, have had a considerable impact on the development of PBL. More recently, the requirements of the 21st century in terms of both knowledge and skills have redefined the needs and roles of both learners and teachers. Presently, PBL embodies a new teaching practice, which models real-life situations for children. It addresses learners’ need to be provided with opportunities to apply their knowledge and skills and enrich their knowledge and improve their skills during activities. Finally, PBL is a method which involves systematic planning (Markham et al. 2003).

Our analysis has explored and compared the perceptions of lower and upper primary and secondary school teachers regarding their roles and teaching activities in PBL and traditional instructional settings. Our results have revealed a teacher preference for group work-based methods such as PBL, work-based learning and cooperative learning. On the other hand, an analysis of the data by school type demonstrated a predominant preference for frontal work, individual work and demonstration often coupled with frontal work in secondary school.

As regards teacher roles, the data suggest that teachers mostly perceive their own roles as motivating, shaping personality and transmitting values. In PBL, controlling students is mostly considered important among beginner teachers. In addition, the roles of teaching, social and affective education and evaluation are relegated to the background during the implementation of projects. In traditional instruction, the teacher roles of motivating learners and transmitting values are also considered important along with the educative role. Thus, we have concluded that teachers perceive themselves as educators rather than facilitators. Maintaining order and discipline is seen as more important in traditional instruction than during project work, where the supervision of students receives very little attention and is mostly addressed by expert teachers. Shaping the community is mostly viewed by teachers as being accomplished in PBL, not in traditional instruction. In conclusion, the results suggest that teachers still strive to play a leading role in the classroom.

We also aimed to gather evidence on the factors that contribute to the success of PBL and traditional instruction. In PBL, a great degree of learner activity and good atmosphere were the most frequently listed, whereas games, the use of ICT tools and good results were not considered vital. In traditional classroom instruction, learner activity, good atmosphere and varied methodology were highlighted. In contrast with the results for PBL, evaluation was foregrounded in the traditional setting. On the other hand, honesty, the role of emotions and games were not mentioned with regard to traditional classroom instruction. Student involvement in evaluation and the methods used play a fundamental role. According to our results, students contribute to evaluation, mostly through oral evaluation. Along similar lines, Van den Bergh et al. (2006) argue for the strategic use of assessment tools to improve learner achievement and the importance of learner perceptions for the interpretation of learning outcomes and various assessment methods, including self- and peer evaluation (Van den Bergh et al. 2006).

Our results provide the option of immediate application as they provide additional guidelines for the development of tools that assess PBL projects more effectively. Furthermore, these findings can be applied to the development of these projects as well as further qualitative and quantitative analyses. Using the full questionnaire, school masters can gain an overview of teachers’ views on PBL. A further option for future research would be to analyse students’ perceptions as well. We also suggest further research on students’ views on their teachers’ roles in this paradigm. Additionally, a more complex evaluation of PBL would provide a comprehensive view from teachers’ and students’ perspectives.
